# Matching optical flow to motor speed in virtual reality while running on a treadmill

**DOI:** 10.1371/journal.pone.0195781

**Published:** 2018-04-11

**Authors:** Martina Caramenti, Claudio L. Lafortuna, Elena Mugellini, Omar Abou Khaled, Jean-Pierre Bresciani, Amandine Dubois

**Affiliations:** 1 Department of Neuroscience and Movement Science, University of Fribourg, Fribourg, Switzerland; 2 Istituto di Bioimmagini e Fisiologia Molecolare, Consiglio Nazionale delle Ricerche, Segrate, Milano, Italy; 3 HumanTech Institute, University of Applied Sciences and Arts Western Switzerland, Fribourg, Switzerland; University of Muenster, GERMANY

## Abstract

We investigated how visual and kinaesthetic/efferent information is integrated for speed perception in running. Twelve moderately trained to trained subjects ran on a treadmill at three different speeds (8, 10, 12 km/h) in front of a moving virtual scene. They were asked to match the visual speed of the scene to their running speed–i.e., treadmill’s speed. For each trial, participants indicated whether the scene was moving slower or faster than they were running. Visual speed was adjusted according to their response using a staircase until the Point of Subjective Equality (PSE) was reached, i.e., until visual and running speed were perceived as equivalent. For all three running speeds, participants systematically underestimated the visual speed relative to their actual running speed. Indeed, the speed of the visual scene had to exceed the actual running speed in order to be perceived as equivalent to the treadmill speed. The underestimation of visual speed was speed-dependent, and percentage of underestimation relative to running speed ranged from 15% at 8km/h to 31% at 12km/h. We suggest that this fact should be taken into consideration to improve the design of attractive treadmill-mediated virtual environments enhancing engagement into physical activity for healthier lifestyles and disease prevention and care.

## Introduction

Human locomotion is guided by multisensory information regarding the relative motion between the body and the environment [1]. This means that visual, vestibular, motor, kinaesthetic and auditory signals are integrated by the central nervous system to give rise to motion perception [2]. Many studies investigated how these signals are integrated for human orientation and path integration [3–11]. Less is known about speed perception. In particular, several studies on speed perception were performed with non-moving subjects and investigated how visual “parameters” like the size and the nature of the field of view, the structure of the visual scene and/or the contrast of visual information affected the perceived speed [12–14]. By contrast, few studies investigated how visual and non-visual/kinaesthetic signals are integrated for speed perception [15–21]. These studies were performed with walking subjects, and they consistently reported an altered perception of visual speed [16, 17, 22–24]. Specifically, when participants walking on a treadmill were asked to match the speed of the optical flow to their actual locomotor speed, they systematically selected a visual speed that was higher than the treadmill speed [16, 17, 22, 24]. In other words, visual speed was underestimated relative to the actual walking speed. However, walking speed represents only a fraction of the speed range of human locomotion. Accordingly, one might wonder whether the results obtained with walking individuals can be generalised to higher movement speeds, as, for instance, those experienced when running. This question is even more legitimate considering that walking and running are distinct gait modes, relying on different mechanical, energetic, and sensorimotor processes.

Walking and running both result from the coordinated action of different muscles. Both gait modes are characterised by the same basic features, each step presenting a stance phase and a swing phase. However, the timing of the events in each cycle differs between the two gait modes. Specifically, the stance phase is longer in walking, whereas the swing phase is longer in running. In addition, whereas at least one foot is always on the ground while walking, running includes a “flight” phase during which neither foot is on the ground. For that matter, the two gait modes have been described through different physical models. Walking is consistent with an inverted pendulum paradigm characterised by a cyclical swing between potential and kinetic energy due to the vertical displacement of the centre of mass of the body. On the other hand, running has been described by the pogo-stick paradigm, which enables the partial recovery of energy stored at each step in muscle/tendon structures with elastic properties [25]. As a result, even at a same locomotor speed, the two gait modes are characterized by different internal loads, such as forces in muscles, bones and ligaments [26, 27]. Along that line, many studies have demonstrated that a specific locomotion pattern is adopted in order to minimise the energetic cost. Specifically, at a critical speed of about 2m/s, the walk-run transition generally ensues. Above that speed, walking no longer requires less energy than running [28–30].

Additionally, studies have evidenced a different modulation of the locomotor pattern in walking and running [31–33]. This is for instance the case for path integration, which is linked to the perception of distance and thus may influence speed perception. Specifically, path integration in running is largely achieved via automated spinal programs that work independently of sensory control. On the other hand, path integration in walking seems to be more dependent on visual control, both afferent and reafferent [34]. Similarly, while vestibular afferents play a great role in walking, there is a central inhibition of these afferents in running, possibly to avoid potentially adverse interactions with the automated motor pattern [31, 34, 35].

Considering the limited speed range of walking as well as the above-mentioned differences between the walking and running gait modes, we investigated how visual and kinaesthetic/efferent information is integrated for speed perception in running. For different running speeds, we measured the optical flow speed that is perceived as matching the actual locomotor/running speed. Specifically, participants ran at different speeds on a treadmill in front of a large screen displaying a moving virtual scene and they were asked to compare the speed of the moving visual scene to their perceived running speed.

## Materials and methods

Twelve moderately trained to trained subjects (4 female, 8 male) with a mean age of 28.5 (±6.0 SD), participated in the experiment. They were naïve about the purpose of the study, had normal or corrected-to-normal vision and none had a history of cardiovascular disease. Participants were either athletes or engaged in sports activities at an amateur level, as assessed through a questionnaire. All participants gave their informed and written consent prior to their inclusion in the study, which was performed in accordance with the ethical standards specified by the 1964 Declaration of Helsinki and approved by the Ethics Committee of the University of Fribourg.

**Fig 1:**
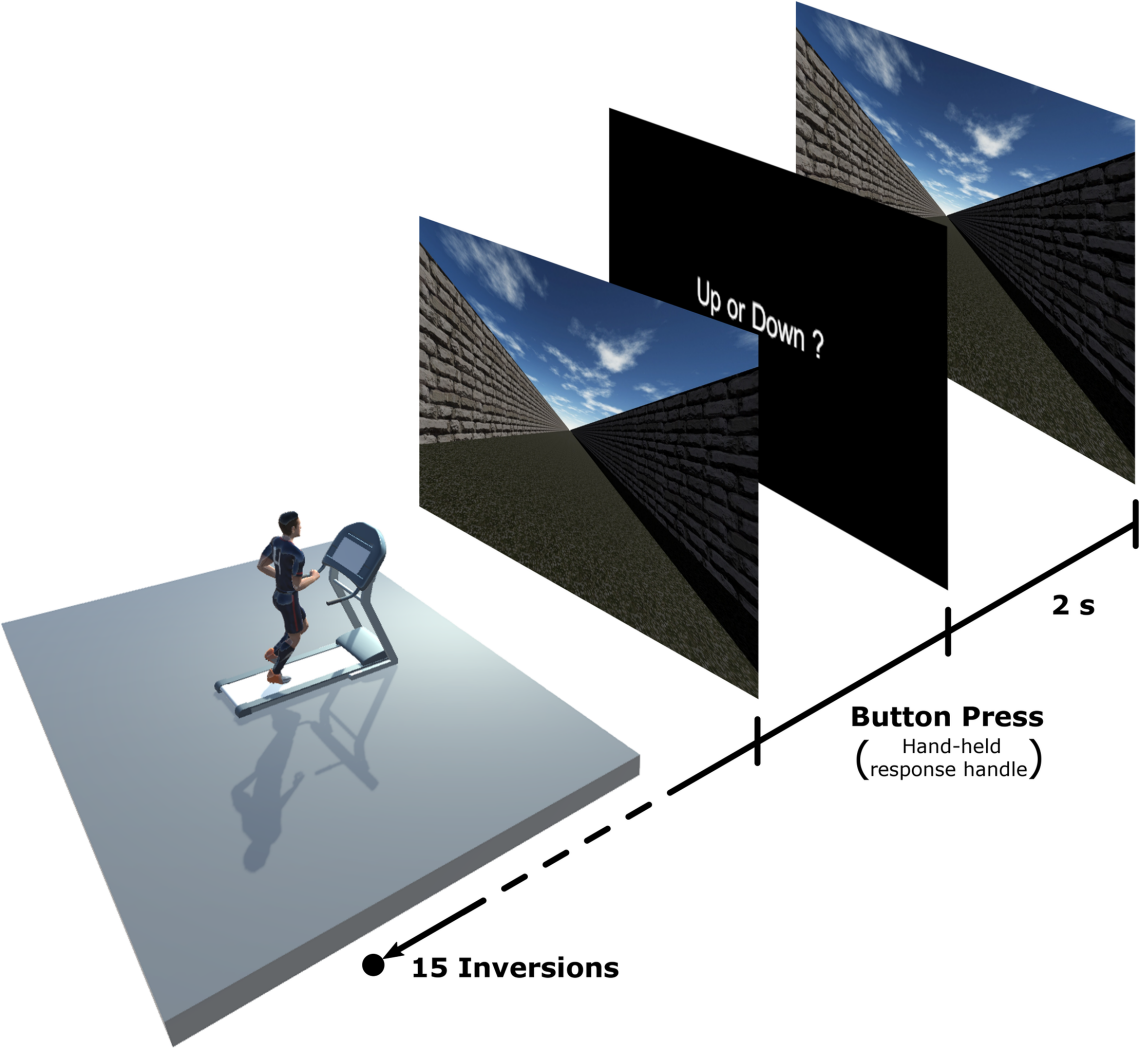
Schematic representation of the experimental set-up. Participants were freely (no hand support) running on the treadmill in front of a large projection screen. For each trial, a moving visual scene was briefly presented and participants were asked to indicate whether the scene was moving faster or slower than their current running speed. Responses were given using handheld cylinders (115 by 30 mm, 15 grams) with a response button on their top surface (not shown in the Figure) and sent via Bluetooth to the computer.

Participants ran on a HP Cosmos Mercury treadmill (Running surface: L: 150cm, W: 50cm) positioned in front of a 4.30m x 2.70m projection screen. To simulate optical flow, a virtual scene created using Unity 3D was projected onto the screen using a Barco F50 WUXGA projector with a 1920x1200 pixels resolution. This virtual scene consisted of an open-air neutral hallway that was presented at constant-velocity motion (see Fig 1). The granular texture of the floor and the random pattern on the walls provided rich optic flow information regarding visual speed, but without any landmarks or usable spatial information. When running, participants’ head was located 256cm from the centre of the screen, which resulted in an effective field of view of 80°. The room was darkened during the experiment, with the display screen being the only source of light. For the whole duration of the experiment, participants ran holding a light (15 grams) custom-made plastic cylinder measuring 115 mm (height) by 30 mm (diameter) in each hand. A response button was located on the top surface of the cylinder, so that participants could effortlessly press on it with their thumb while running, using the switch held in the left hand to increase the speed of the visual scene and the switch held in the right hand to decrease it. The responses were sent via Bluetooth to the computer.

The experiment consisted of three blocks. In each block, the participants ran at one of three different speeds (i.e., treadmill speed): 8km/h (2.23m/s), 10km/h (2.78m/s) or 12km/h (3.34m/s). The order of presentation of the three running speeds (i.e., the blocks) was randomized and counterbalanced between subjects. While running, the participants were presented with successive short sequences of the visual scene. Each sequence was presented for two seconds, and its speed changed from trial to trial (i.e., from one sequence to the next), being independent of treadmill speed. Participants were instructed to estimate if the scene was moving slower or faster than the actual running speed. For each running speed, four consecutive tests were proposed in a random order using a 1-up-1-down staircase method [36, 37]. The staircase started two times with a higher (+4km/h (+1.11m/s)) and two times with a lower (-4km/h (-1.11m/s)) visual speed than the actual treadmill speed. The speed of the visual scene was adjusted with the staircase method, which defined increases/decreases of the visual speed by steps of 0.5km/h (0.14m/s) until the first inversion of the response, and then by steps of 0.3km/h (0.08m/s). This method allowed us to determine the Point of Subjective Equality (PSE), which is a perceptual threshold indicating the respective values of two quantities when they are perceived as equivalent. In our case, for each participant and each of the three running speeds, PSE indicated to us what speed of the visual scene–i.e., optical flow–was perceived as matching the treadmill speed. In other words, perceived visual speed was not an absolute speed value, but rather corresponded to the perceptual matching between visual speed and running speed.

At the beginning of the experiment and prior to the testing phase, the participants spent a few minutes familiarising themselves with treadmill running at different speeds. They were then familiarised with the experimental setting, response switches and task, using a training program, which presented the visual scene at different speeds. Experimental trials were then initiated when the participant felt comfortable running at different speeds. The single trials of each test started with the participant running at constant speed. Participants were instructed to gaze toward the end of the virtual hallway. The visual scene was presented for 2 seconds before the participants were challenged with a black screen presenting the question “Up or down?”:

- “Up” if the visual speed appeared slower than their running speed, so that they wished to increase the speed of the visual scene
- “Down” if the visual speed appeared faster than their running speed, so that they wished to decrease the speed of the visual scene.

Participants gave their response by pressing with their thumb on the response button of the cylinder held in their left (Up) or right (Down) hand. Once the participant pressed the response button, the visual scene of the following trial was displayed on the screen. Each staircase ended when 15 inversions of the responses were reached. At the end of each staircase test, the participants could take a short pause before starting the next test. The duration of each staircase test depended on the number of trials needed to reach 15 inversions of the responses. On average, this took 84s at 8km/h, 88s at 10km/h and 101s at 12km/h.

## Results

For each of the three treadmill speeds (i.e., 8, 10 and 12km/h), we compared the perceived visual speed with the actual running speed–i.e., the speed of the treadmill–using in each case either a t-test (when perceived visual speed data was normally distributed, as assessed using the Shapiro-Wilk test) or a Wilcoxon signed rank test (when perceived visual speed data was not normally distributed). For all three tests, the alpha level was corrected for multiple comparisons using Bonferroni correction (i.e., 0.05/3).

**Fig 2:**
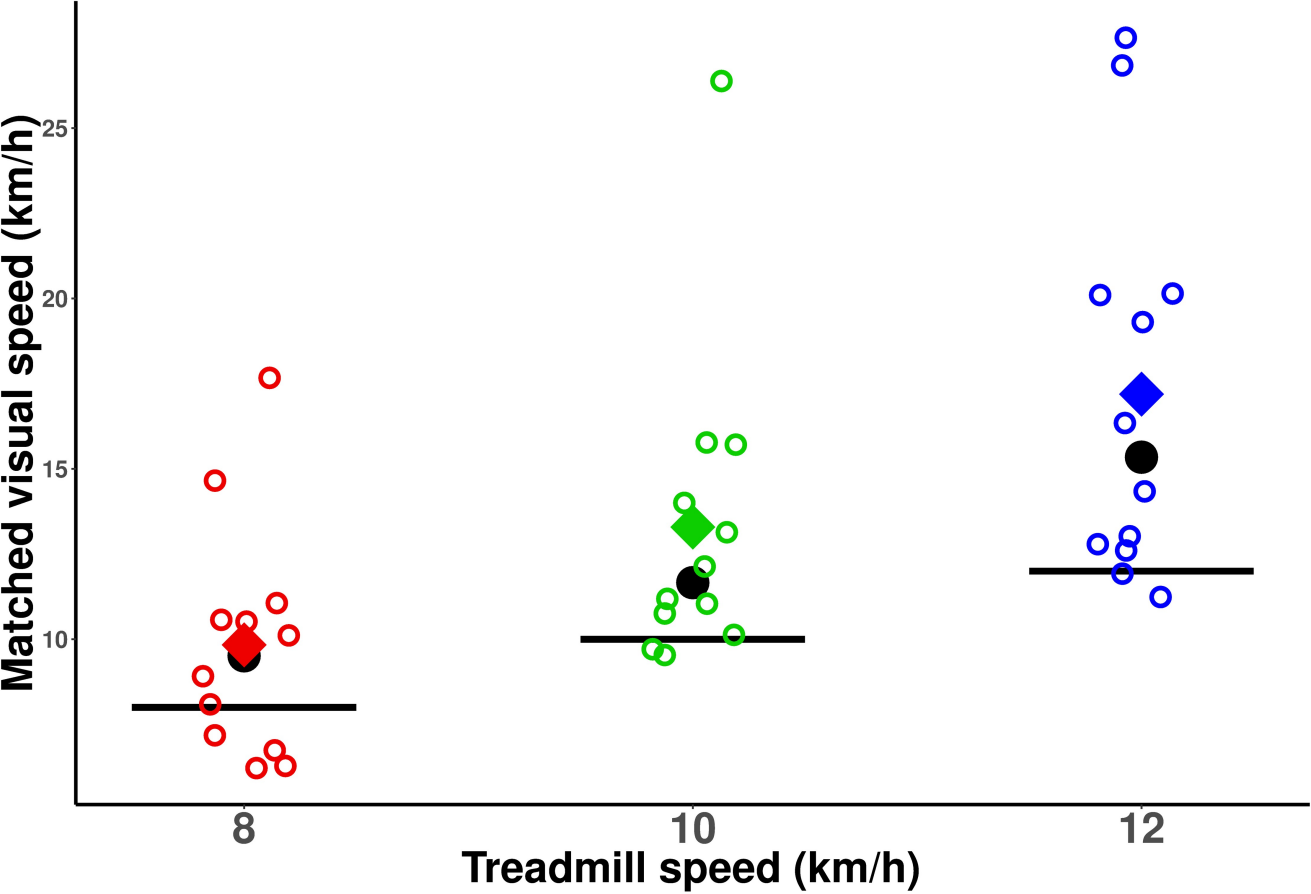
Speed of the visual scene that was perceived as matching running / treadmill speed. For most participants (empty circles) and on average (filled diamonds), the visual scene needed to move faster than the actual running speed (black lines) to be perceived as equivalent. The black circle corresponds to the median value.

For all three treadmill speeds, participants set a visual speed that was higher than the actual treadmill speed (see Fig 2). In other words, the optical flow had to move faster than the actual running speed for the two speeds to be perceived as matching. This means that visual speed was systematically underestimated relative to running speed. This underestimation was significant both for a 10km/h (Wilcoxon, V = 73, p<0.01) and 12km/h (t-test, t(11) = 3.17, p<0.01) treadmill speed, but failed to reach significance for a treadmill speed of 8km/h (t-test, t(11) = 1.825, p = 0.095).

**Fig 3:**
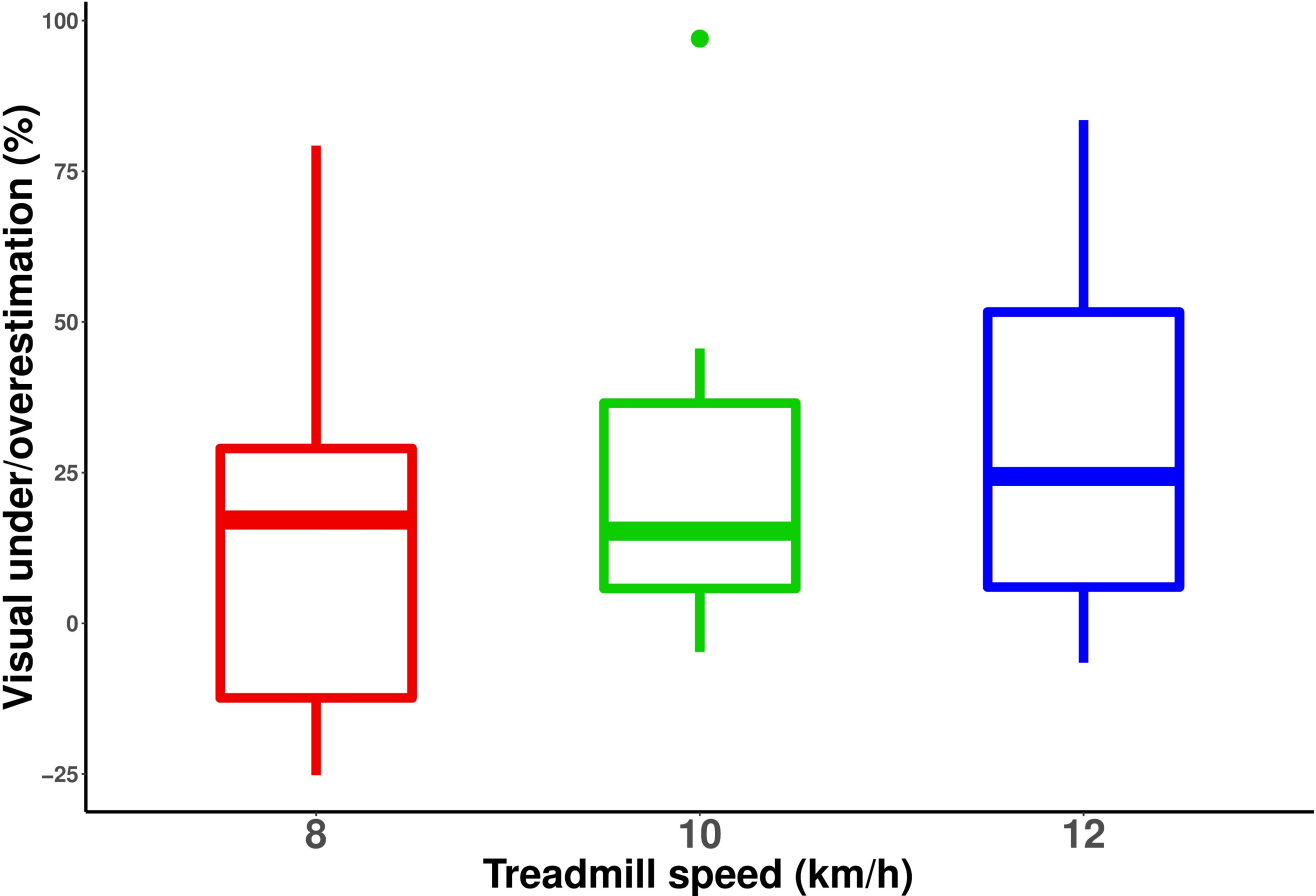
Percentage of underestimation (positive values) and overestimation (negative values) of visual speed relative to running speed. Estimations were computed using the equation: ln(perceived visual speed / actual treadmill speed) * 100. Each box summarizes the distribution of responses of all participants. The central line corresponds to the median, the box defines the inter-quartile range (IQR, between first and third quartile), and the whiskers correspond to ±1.5 IQR.

We then tested whether visual underestimation was of similar amplitude for all three treadmill speeds. For each treadmill speed, we computed the percentage of visual under/overestimation using the following equation: ln(perceived visual speed / actual treadmill speed) * 100. This percentage indicated how much faster–for positive values–or slower–for negative values–the optical flow had to move relative to the treadmill speed for the two speeds to be perceived as equivalent. We used a logarithm (i.e., *ln*) so that neither visual nor treadmill speed constituted the absolute reference value [38]. Fig 3 shows these percentages for all subjects and all three treadmill speeds. A Friedman rank sum test (as data was non-normally distributed) was used to compare the percentage of underestimation between the three treadmill speeds. The test indicated that this percentage differed significantly between the three treadmill speeds (chi-squared(2) = 7.1667, p<0.05). In particular, visual underestimation was significantly larger for a treadmill speed of 12km/h (average underestimation of 31.33%) than for a treadmill speed of 8km/h (average underestimation of 15.46%).

## Discussion

Participants running on a treadmill in front of a moving virtual scene were asked to match the visual speed of the scene to their running speed–i.e., treadmill speed. For all three running speeds tested in the experiment, namely 8, 10 and 12km/h, the visual speed of the virtual scene was systematically higher than the actual running speed in order to be perceived as equivalent to treadmill speed. In other words, participants systematically underestimated the visual speed relative to their running speed. This underestimation was speed-dependent, as the percentage of underestimation relative to running speed increased with treadmill speed.

### Integration of optical flow information in running vs. walking

Even if walking and running can be considered as distinct gait modes, the underestimation of visual speed–relative to locomotor speed–observed in the current study with running individuals is similar to that reported in previous studies performed with walking individuals [16, 17, 22, 24]. Specifically, for both running and walking subjects, the actual locomotion speed was systematically matched to a faster-moving visual scene. Because all studies investigating visual-kinaesthetic integration for speed perception in locomotion were based on virtual visual scenes, this systematic underestimation of visual speed could be related to a misperception of distance. In fact, research has shown that people tend to underestimate egocentric distance in virtual environments, objects and landmarks being usually perceived as closer to the viewer than they actually are [3, 39–42]. Because speed corresponds to the distance covered in a given time, if distance is misperceived, this should logically affect speed perception. In particular, if visual distance is underestimated, then visual speed should also be underestimated, because subjects would get the perception of having covered a shorter distance in the same amount of time.

If visual speed is systematically underestimated relative to locomotion speed both for walking and running, there are nonetheless differences between the visual-locomotor gain (i.e. *visual speed / treadmill speed*) measured in the current studies with running individuals and those reported in previous studies with walking individuals. For treadmill walking, this gain varies among studies. For instance, Powell [22] found visual-locomotor gains ranging from 1.52:1 to 2.41:1, with a mean of 1.93:1, whereas other authors have reported gains ranging from 1.15 to 1.46 [17, 43]. Here, with running individuals, we found visual-locomotor gains ranging from 0.78:1 to 2.64:1, with means ranging from 1.23:1 to 1.43:1, depending on the running speed. This is slightly higher than what has been reported for most studies on walking individuals. In addition, whereas visual-locomotor gains observed with walking individuals remained constant across all walking speeds [22, 43], in the current study with running individuals, we observed a speed-dependent underestimation of the visual speed. Precisely, visual-locomotor gain increased with increasing running / treadmill speeds, with a mean gain ranging from 1.23:1 at a running speed of 8km/h, to 1.32:1 at 10km/h and 1.43:1 at 12km/h. This may suggest that during exercise at high velocity, factors associated with the intensity of motor activation (such as proprioceptive stimuli) and/or with metabolic response to exercise (such as blood lactate levels or neural autonomic activation) play a role in the perception of locomotor speed. Such influence of motor effort on visual perception would be consistent with previous studies that have evidenced effort-evoked biases in the judgement of self-relative gait speed, slant and distance [44–46]. Along that line, we should mention here that some studies have shown that when fatigue kicks in, runners tend to change running kinematics in an attempt to maintain running speed [47, 48]. When running overground, reducing the running speed has been suggested to constitute a way to prevent injuries [49, 50]. Such a speed reduction was obviously not possible in our experiment because of the speed constraint imposed by the treadmill, which possibly influenced step variability and the energy cost of running [51].

The discrepancy between the visual-locomotor gains observed in our study and those reported in previous studies on walking could also result from methodological differences, notably regarding the type of display used to present the moving visual scene. In particular, whereas in Banton et al. [16] and Durgin et al. [17, 43] the visual scene was displayed using a Head-Mounted Displays (HMD), in our study we used a 4.30m x 2.70m projection screen, similar to the 4.5m x 2m projection screen used by Powell et al. [22]. It is important to note here that this latter study also found relatively high visual-locomotor gains. A second “display-related” difference lies with the size of the horizontal field of view (FoV), which was narrower than 60 degrees in Banton et al. [16] and Durgin et al. [17, 43] but of 80 degrees in our study and 100 degrees in Powell et al. [22]. Incidentally, the studies in which the FoV was narrower are also the studies in which a HMD was used. However, previous studies on visual speed perception have shown that a more important stimulation of the peripheral portion of the FoV is associated with an increase of perceived visual speed [12, 13]. Consequently, a larger FoV should give rise to an over- rather than an underestimation of visual speed. Therefore, if the underestimation of visual speed observed in the current study has a display-related component, it is more likely to result from the type of display used, namely HMD vs projection screen, than from the size of the FoV.

### Specificity of treadmill vs. overground locomotion

In addition to the abovementioned factors, which are mostly vision-related, other kinaesthetic-related factors could also have affected speed perception. In particular, treadmill running by itself has been shown to influence speed perception. Precisely, locomotion speed on treadmill is usually perceived as higher than the corresponding overground speed. For instance, Kong et al. [52, 53] found that when asked to run consistently at their preferred speed both overground and on a treadmill, participants were unable to match the overground speed when running on the treadmill. On average, participants ran 27.1% slower on the treadmill, even though they thought to be running at the same speed. This fact is in accordance with previous studies on treadmill walking, in which participants felt to be walking faster on the treadmill, even though the speed was nearly equivalent to overground trials [54]. Similarly, participants asked to walk at a speed corresponding to a given rate of perceived exertion (RPE)– 13 on a scale of 6 to 20 –walked slower on treadmill than overground [55]. All these results highlight a systematic overestimation of locomotor speed on treadmill. A similar overestimation of locomotor speed might also have played a role in the results observed in our study. Indeed, an overestimation of treadmill speed would directly affect the gain between perceived locomotor speed and perceived visual speed, precisely in the direction that we measured in our study.

Along a similar line, differences in running kinetics and kinematics for overground and treadmill running have been reported to influence the capacity to discriminate running speed [56–59]. Specifically, high-speed cinematography showed longer periods of support, smaller vertical velocity of the centre of mass, less variation in the horizontal and vertical velocities of the centre of mass, shorter stride length and higher stride rate for treadmill compared to overground running [56, 60, 61]. Also, some studies have shown that for walking, the ratio of step length to step frequency tend to be constant for most individuals even over large changes in walking speed [62]. This walk ratio is lower during treadmill walking, due to a higher step frequency relative to stride length. According to Durgin et al. [43], the higher step frequency on treadmills might represent a source of bias in the perceived speed of treadmill walking since step frequency normally dominates in the estimation of motor walking speed [20, 62–64]. Similar alterations in stride length and stride rate during treadmill running, with shorter and more frequent steps as compared to overground running would also likely bias perceived locomotor speed.

Unfamiliarity with treadmill running [65] might also have contributed to the altered perception of running speed that we observed. In particular, differences in balance and coordination [66], increased attentional demand [67] and fear to fall off the treadmill could all have played a role in speed overestimation. As compared to previous studies that investigated perceived visual speed during treadmill locomotion, this latter aspect might have played a more important role in our study because no fixed handrails for support were provided. We made this choice so that participants could freely and naturally move their upper limbs, as in overground running. Yet, most abovementioned studies [16, 17, 43] but that of Powell et al. [22] provided participants with fixed handrails to help them stabilize themselves during locomotion. In addition to have possibly increased felt safety, such handrails provided an additional and fixed haptic feedback which probably contributed to increase the incongruence between locomotor information about movement and haptic information about lack of mobility. It is not clear to which extent hand support, when supplied, might have affected self-motion perception, but as a matter of fact, the studies in which no hand support was provided were those in which the underestimation of visual speed was the largest.

### Implications for the design of treadmill-based virtual environments

The use of virtual environments for treadmill activities could reduce the abovementioned differences between overground and treadmill running. It would notably contribute to minimize the discrepancy between kinaesthetic / motor and visual information. During real overground walking and running, a complex of forces is generated by the skeletal muscle system and applied onto the ground to ultimately produce a forward movement. The relative motion between the moving individual and the stationary surrounding environment generates an optical flow. During treadmill walking and running, similar biomechanical forces are produced but without any physical translation with respect to the surrounding environment. This lack of optical flow induces a discrepancy between kinaesthetic / efferent information and visual information. In that context, virtual reality may play an important role in enriching the subjective perception of exercise by providing appropriate visual information of movement. An obvious step in that direction would be to display an optical flow that is perceived as consistent with kinaesthetic and efferent information of movement. One could notably imagine providing personalized gains between treadmill and optical flow, for instance by using pre-workout quick calibration routines that would allow the user to determine which visual gain gives rise to the most “natural” visual feedback.

Treadmills constitute one of the most commonly used pieces of equipment to train cardio-vascular fitness indoor. One of the major disadvantages of treadmill activity is that it tends to be boring and monotonous as compared to outdoor activity [68]. To palliate this monotony, most gyms and fitness clubs provide TV and music for a more pleasant experience during training. However, treadmill-mediated virtual environments could even further enhance the user’s engagement, and provide a more enjoyable and stimulating experience. Notably, they might provide the runner / walker with different types of virtual outdoor environments, which would for instance allow the user the walk or run in the streets of New York City, London, Rome, or in the Serengeti Natural Park. This, by producing subjective enjoyment and satisfaction, would likely increase engagement/adherence to physical activity, which is an important achievement in the context of health and diseases prevention.

## Supporting information

S1 Dataset Collection of recorded data. (CSV)
